# Modeling Pitch Perception With an Active Auditory Model Extended by Octopus Cells

**DOI:** 10.3389/fnins.2018.00660

**Published:** 2018-09-25

**Authors:** Tamas Harczos, Frank Markus Klefenz

**Affiliations:** ^1^Fraunhofer Institute for Digital Media Technology, Ilmenau, Germany; ^2^Auditory Neuroscience and Optogenetics Laboratory, German Primate Center, Goettingen, Germany; ^3^Institut für Mikroelektronik- und Mechatronik-Systeme gGmbH, Ilmenau, Germany

**Keywords:** auditory modeling, latency-phase coding, inter-spike interval histogram, time domain parameterization, pitch, pitch estimation, octopus neuron, Hough-transform

## Abstract

Pitch is an essential category for musical sensations. Models of pitch perception are vividly discussed up to date. Most of them rely on definitions of mathematical methods in the spectral or temporal domain. Our proposed pitch perception model is composed of an active auditory model extended by octopus cells. The active auditory model is the same as used in the Stimulation based on Auditory Modeling (SAM), a successful cochlear implant sound processing strategy extended here by modeling the functional behavior of the octopus cells in the ventral cochlear nucleus and by modeling their connections to the auditory nerve fibers (ANFs). The neurophysiological parameterization of the extended model is fully described in the time domain. The model is based on latency-phase en- and decoding as octopus cells are latency-phase rectifiers in their local receptive fields. Pitch is ubiquitously represented by cascaded firing sweeps of octopus cells. Based on the firing patterns of octopus cells, inter-spike interval histograms can be aggregated, in which the place of the global maximum is assumed to encode the pitch.

## Introduction

Sensation of pitch is a perceptual category. Pitches are for instance reproducibly generated by music instruments or singing voices, and are notated in musical notes. Each note is assigned a fundamental frequency F0 by reference to the root tone and tuning system. In addition, pitch sensations are evoked by tonal audio data segments as sinusoids, or sinusoids with resolved and unresolved harmonics (even in the case of missing fundamental frequency), and iterated ripple noise (Huang and Rinzel, 2016). Computational pitch models need to be able to generate pitch hypotheses, which can be compared to the annotated ground truth of the audio source data. Various computational pitch models have been compared in a common evaluation matrix and transparently benchmarked in international open contests (Downie, 2008; Cunningham et al., 2017).

Models of pitch perception have been created, implemented and discussed for the auditory system (Oxenham, 2013, 2018; Laudanski et al., 2014; Langner, 2015; Friedrichs et al., 2017; Tang et al., 2017). The two classic pitch models of the auditory system are based either on place or temporal coding. In the first case pitch is solely dependent on the position of an activated characteristic fiber (CF) along the cochlea. It is a pure place code by indexing the innervated auditory nerve fiber (ANF) along the tonotopically ordered axis. In the second case pitch is derived from inter-spike interval histograms of consecutively firing CFs relying on phase locking of the auditory nerve spike firings to quasi-stationary tonal signals (Stolzenburg, 2015; Joris, 2016). Neuro-physiologically parameterized auditory models mimic the dynamics of the basilar membrane, the mechano-electrical coupling of inner hair cells, and the membrane voltage regulated vesicle rate-kinetics (Voutsas et al., 2005; Balaguer-Ballester et al., 2009). Several pitch decoders are constructed as neural networks (Ahmad et al., 2016; Barzelay et al., 2017). Some recent pitch decoders are realized as spiking neural networks in which Spike-Timing Dependent Plasticity (STDP) learning rules are applied (Saeedi et al., 2016, 2017). STDP is ubiquitous in contemporary computational neuroscience and a plethora of variations exist as for instance spike triplet rules imitating the NMDAR/AMPAR opening/closing cascades (Shahim-Aeen and Karimi, 2015; Krunglevicius, 2016; Acciarito et al., 2017; Amirsoleimani et al., 2017; Zeng et al., 2017). Several STDP learning rules with and without synaptic, dendritic, somatic and axonal delays have recently been formulated (Susi, 2015; Taherkhani et al., 2015; Sun et al., 2016; Asl et al., 2017; Bagheri et al., 2017; Chrol-Cannon et al., 2017; Fu et al., 2017; Matsubara, 2017; Miró-Amarante et al., 2017; Panda et al., 2017; Tavanaei and Maida, 2017; Xie et al., 2017; Yin et al., 2017).

The perception of pitch for cochlear implant (CI) users is an urgent open research topic, because implantees often don't profit from music entertainment, as music is sometimes perceived as an unpleasant impression. CI users often can resolve pitch poorly by mismatching it by several half-tones (Harczos et al., 2013a) in comparison to normal hearing listeners where just-noticeable differences (JNDs) in the frequency of a pure tone are as low as 0.2% for well-trained listeners in the mid-frequency range of 500 Hz to 2 kHz (Moore, 1973).

Even applied temporal fine structure (TFS) CI strategies for pitch perception are felt unsatisfactory (D'Alessandro et al., 2018). One causal source of the CI limitations is the inevitable current spread of the electrodes, which leads to an excitation volume, in which several ANFs are excitable in contrast to the point to point interconnections between inner hair cells and spiral ganglion cells via the synaptic boutons (Jürgens et al., 2018). Biesheuvel et al. (2016) analytically discuss the relation between the excitation density profile (EDP) of the electrodes and the spread of excitation (SOE). Another perceptual obstacle for implantees are the distortions caused by frequency misalignment related to the expected vs. real electrode positions (Marozeau et al., 2014; Seeber and Bruce, 2016; Jiam et al., 2017). Several investigations for improvements of pitch perception for CI users have been made by concise variations of stimulation patterns, stimulation rates, number of electrodes, insertion angles and frequency allocation maps (Kalkman et al., 2014; Schatzer et al., 2014; Hochmair et al., 2015; Landsberger et al., 2015; Devocht et al., 2016; Marimuthu et al., 2016; Rader et al., 2016; Todd et al., 2017).

If we better understand how the peripheral nervous system senses and constitutes pitch as a categorical entity, CI strategies with better pitch signaling can be devised.

The active auditory model of SAM generates cochleagrams with characteristic repetitive latency-phase trajectories (Harczos et al., 2013b). Our proposed pitch decoder model is based on decoding these repetitive latency-phase trajectories by octopus cells, whose repetitively firing translates to inter-spike intervals, which accumulate to inter-spike interval histograms (ISIHs). The latency-phase trajectories are covered by overlapping local receptive field patches of the ensembles of octopus cells which fire upon the local detection of a segment of a latency-phase trajectory. The time-reciprocal of the global maximum of all octopus ISIHs is assumed to be the found pitch. The model is tested and evaluated by analyzing pitch from tones of various sources.

## Methods

Knowing the fundamental frequency of a signal is often a prerequisite for further processing of acoustic signals, no matter if it is used for complex tasks like automatic music transcription, or just as supporting information for e.g., speech compression or gender identification (Strömbergsson, 2016). A plethora of F0 estimators have already been reported and discussed (Jouvet and Laprie, 2017; Stone et al., 2017). Often cited F0 estimators are Praat (Martin, 2012), YIN (De Cheveigné and Kawahara, 2002), and RAPT (Talkin, 1995), among others. An international community was established in 2005 to annually benchmark F0 estimation methods and report the state of the art achievements (MIREX, 2018). In MIREX, currently YIN is used as the golden standard for the annotated ground truth. The actual state of the art is given in the MIREX 2017 survey report: “Multiple Fundamental Frequency Estimation and Tracking Results” (MIREX, 2017).

Our method can't currently keep up with most of the contestants of MIREX, as it would still need additional parts like a multiple F0 separator and a melody contour segmenter, as given for example in (Ycart and Benetos, 2018). Rather, we would like to show a bio-plausible way of F0 estimation as a possible starting point for novel research, along with first results for solo instruments and singers, to get an impression of its quantitative performance.

**Figure 1:**
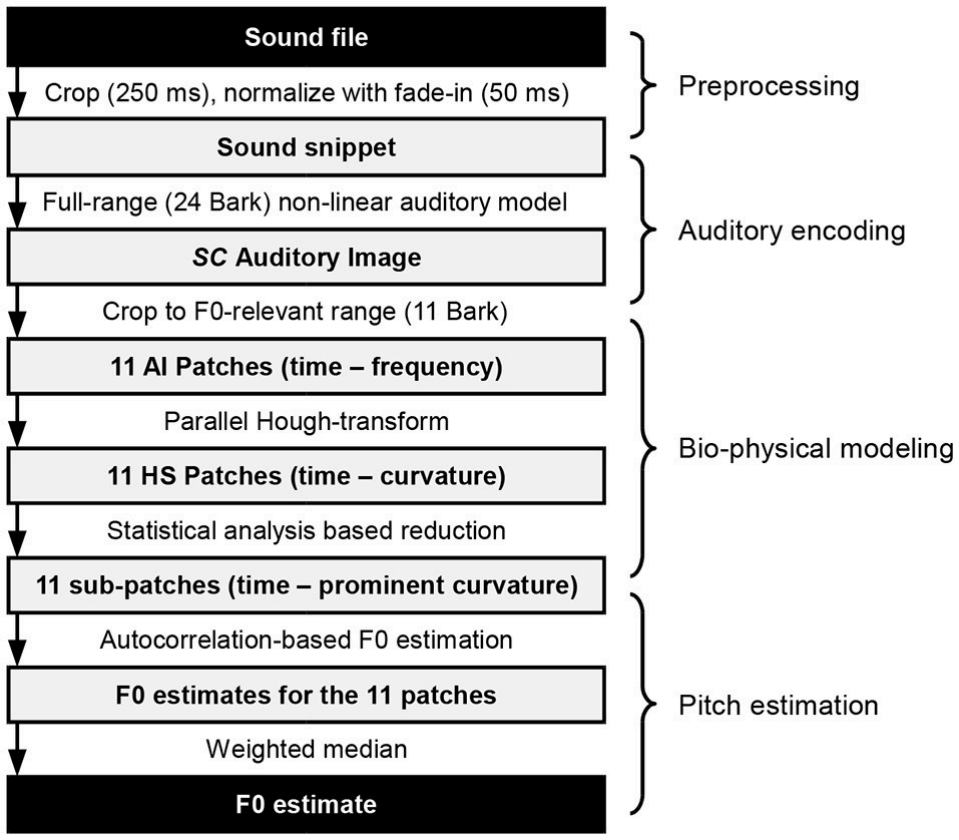
Overview of the processing steps from a single sound file to the pitch estimate.

A chain of concatenated processing steps leads to the final estimation of F0. These are realized as computational blocks, and can be categorized into preprocessing, auditory encoding, bio-physical modeling and pitch estimation, as shown in Figure 1. Their inner workings are presented throughout the following sections.

### Test corpora and preprocessing

For testing the presented system we used with three kinds of sounds: pure tones, sung vowels (*a:* and *i:*, sung by a female as well as a male singer), and solo instruments (violin, flute, and piano). The latter were taken from the MUMS (McGill University Master Samples) CDs (Opolko and Wapnick, 1987) and correspond to CD1 Track6 (Violin, bowed), CD2 Track5 (Alto flute), and CD3 Track3 (9′ Steinway grand piano, plucked). The sung vowel database was created at the Fraunhofer Institute for Digital Media Technology IDMT and can be obtained free of charge by contacting the authors.

Each input file has been presented to the auditory encoder as mono signal, sampled at 44,100 Hz and 16-bit resolution. In the preprocessing stage a 250 ms long snippet is cropped from the input sound file (For the data presented in this paper, we aimed to extract the middle part of each sound file). Next, the sound snippet's amplitude is normalized to yield around 65 dB SPL in the subsequent auditory model. Finally, a 50 ms long linear fade-in is applied to the snippet.

### Auditory encoding

SAM is a cochlear implant sound processing strategy based on a neuro-physiologically parameterized model of the peripheral hearing (Harczos et al., 2013a). SAM's auditory model can be categorized as a transmission-line model augmented with the contractive electro-motility by outer hair cells, and the basilar membrane coupled to inner hair cell rate-kinetics. The transformational process cascades from sound conversion up to cochleagrams of parallel spike trains along the auditory nerve are modeled by structured generative modules, which are ruled by physical equations and their numerical solutions. SAM is basically composed of a sound triggered basilar membrane movement solver part and a spike generation model part of the innervated auditory nerve. For the purpose of modeling the basilar membrane movement, the basilar membrane is split into equally long sections and the hydro-mechanical process of vibrational induction is formulated by partial differential equations (Baumgarte, 1997). The mass and stiffness of the cochlear partitions are transposed to their electrical equivalents and the electrodynamic equations are numerically solved by a computer program. The outer hair cell function is described as an electrical feedback loop (Baumgarte, 1997). Inner hair cells (IHCs), which are aligned equidistantly along the cochlea couple to the basilar membrane motion. The fluid movements drive the displacements of the stereociliae of the inner hair cells. The displacement is modeled by forced harmonic oscillator equations. The displacements of the stereociliae induce releases of neurotransmitters in the synaptic clefts (SC) between inner hair cells and the associated spiral ganglion neurons (SGNs) of the auditory nerve. The sound induced time varying cleft concentrations are modeled by Ca^2+^ rate-kinetic equations explicitly given by an analytic IHC computer simulation model (Sumner et al., 2002). The excitatory postsynaptic potentials (EPSPs) of the SGNs are proportional to the ion channels opened and hence proportional to the neurotransmitter concentrations in the synaptic clefts.

**Figure 2:**
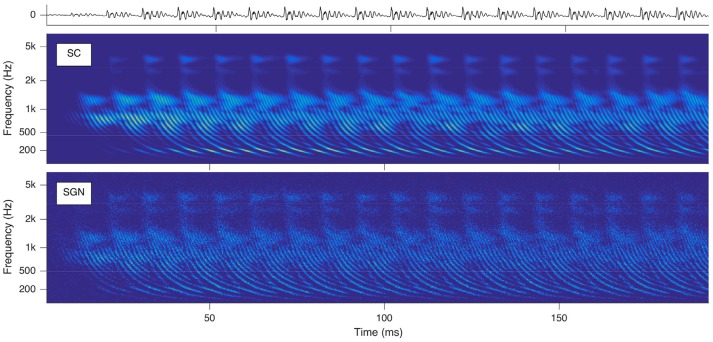
Cochleagrams with quasi-stationary repetitive patterns for a short snippet of the vowel *a:*, sung by a male singer at the note of G2. **Top**: sound signal waveform. **Middle**: probability (ascending from blue over green to yellow) of neurotransmitter substance release into the synaptic cleft as a function of time and place within the cochlea. **Bottom**: action potentials of the spiral ganglion neurons. Note that the ordinate shows the characteristic frequency of the basilar membrane model at the corresponding cochlear position.

The SGNs spike as soon as the exciting EPSPs reach their depolarization thresholds. Hence time-varying audio signals are idiosyncratically transformed into their cochleagram representations instantiated by parallel spike trains of the auditory nerve cells topologically numbered from their locations between round window and apex in ascending order and the times of spike occurrences. Sounds trigger characteristic basilar membrane movements, which appear as traces of delay trajectories of hyperbolic shape in the cochleagram (see Figure 2). The physical reason is the hyperbolically decaying dispersion of the traveling waves along the basilar membrane, slowing down from the base alongside to the apex due to a softer stiffness and heavier mass of the basilar membrane. These repetitively occurring delay trajectories serve as pitch cues.

All further calculations reported in this paper are based on the *SC* modeling stage. The reason is that the calculation of this stage is computationally less demanding, while the results retain all properties relevant for further processing.

### Bio-physical modeling

Auditory nerve fibers project to octopus cells in the ventral cochlear nucleus (VCN). Octopus cells, in turn, project to the superior paraolivary nucleus (SPON) and to the columnar area of the ventral nucleus of the lateral lemniscus (VNLL; Oertel et al., 2017; Felix et al., 2018). Octopus cells are named for their miniature resemblance to octopus with dendrites emanating unidirectionally rostralward from the cell body (McGinley et al., 2012). The dendrites of octopus cells lie perpendicular to the tonotopically organized array of ANFs and therefore their receptive fields are given by their targeted interconnections to the ANFs (McGinley et al., 2012). Each octopus cell receives input from at least 60 ANFs (Spencer et al., 2012). Individual octopus cells experience a local segment of the traveling wave delay, because their receptive fields extend only over a part of the tonotopic axis of the cochlea. Many small synaptic inputs must sum to generate the large synaptic current necessary to evoke an action potential. Octopus cells detect the coincident activation of groups of ANFs by broadband transient sounds with remarkable temporal precision (Golding and Oertel, 2012). Octopus cells rectify latency-phase trajectories in their local receptive fields by dendritic electrotonic filtering of broadband transient sounds in compensating for cochlear traveling wave delays (McGinley et al., 2012). Their tuning will be individually estimated from their location along the tonotopic axis, whereas their individual firing behavior to broadband transient sounds can be simulated in the time domain (Werner et al., 2009). Below 800 Hz, octopus cells generally produce an action potential in response to every cycle of the tone, and above 2 kHz, octopus cells produce a single action potential at the onset of the tone, with no subsequent spikes (Spencer et al., 2012).

In a predecessor model, latency-phase trajectories were globally identified by applying a hyperbolic Hough-transform covering the full ANF range (Harczos et al., 2006). Local maxima in the hyperbole-time space represent their corresponding latency-phase trajectories. For pitched quasi-stationary audio inputs these maxima occur repetitively. Pitch is easily resolved in this model as the inverse of the time interval between two consecutive local maxima aligned along a common hyperbole.

For the presented work, the global model has been refined in several ways to become more bio-compatible. The global Hough-transform is substituted by local parallel Hough-transforms in patches restricted by the number of ANF inputs. Each local patch is analyzed by an ensemble of dedicated octopus cells. Each octopus cell is tuned for a specific local hyperbolic shape section and is therefore part of the distributed Hough-transform execution.

Although the auditory encoder processes the input audio signal in a full 24 Bark frequency range, we restrict our model of pitch estimation for demonstration purposes to frequencies between *F*_*min*_ = 75 Hz and *F*_*max*_ = 1,500 Hz representing roughly 50 semi-tones, spanning a total bandwidth of about 11 Bark. Consequently, we work with eleven patches, whereas every one of them represents the neurotransmitter release probability (as a function of time and cochlear position) for an ensemble of inner hair cells within a frequency-specific region of the basilar membrane corresponding to 1 Bark. The patches are partly overlapping, and for each of them several octopus cells are dedicated to cover several trajectories with diverging local curvatures depending on their positions along the ANFs.

For an *N* sample long audio signal sampled at frequency *f*_*s*_, the neurotransmitter release probability near the *i*-th simulated inner hair cell will be noted *p*_*i*_[*n*], for *n*∈{1,2, …, *N*}. Then, the *k*-th auditory image (AI) patch composed of the AI channels *u* to *v* (where u < v and higher channel number corresponds to higher characteristic frequency) can be noted as:

(1) $$P_{\left(u,v\right)}^{AI}\left[t\right]=P_{k}^{AI}\left[t\right]=\left[\begin{array}{c} \;p_{v}\left[t\right]\\ \ldots \\ \;p_{u+1}\left[t\right]\;\\ \;\;p_{u}\left[t\right]\; \end{array}\right]\;,\;\;t\in \left\{\frac{1}{f_{s}},\;\frac{2}{f_{s}},\;\ldots ,\;\frac{N}{f_{s}}\right\}\;.$$

**Figure 3:**
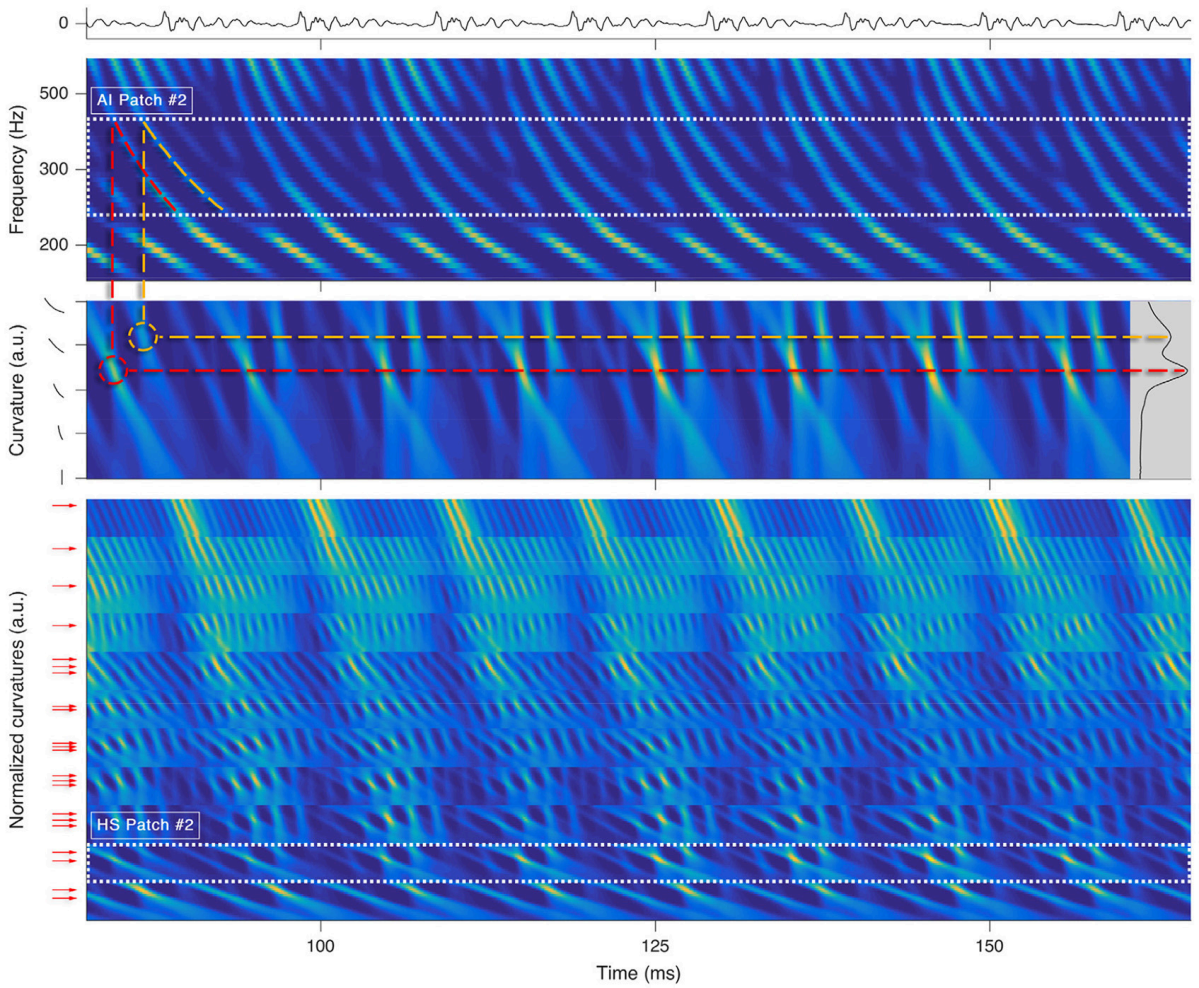
Processing stages for a short snippet of the vowel *a:*, sung by a male singer at the note of G2. **Top**: sound signal waveform. Second row: a zoom-in on the SC auditory image (AI). The range corresponding to $P_{2}^{AI}$ is highlighted by dots. Third row: $P_{2}^{HS}$, i.e., the Hough-space (HS) time-aligned with the input of the parallel Hough-transform for $P_{2}^{AI}$. The inset on the right shows the variance along the time axis for all possible curvatures. Dashed lines illustrate the link between prominent trajectories in the SC auditory image and the local maxima in the Hough-space. **Bottom**: All HS patches stacked onto each other. The range of $P_{2}^{HS}$ is highlighted by dots. The arrows left to the ordinate correspond to detected prominent curvatures *C*_*k*_,_*j*_. By keeping only the rows pointed by the arrows within each of the patches, we yield the sub-patches, which will be used for the F0 estimation in the next processing step.

Each octopus cell rectifies a local trajectory segment in its receptive field by compensating the traveling wave delay (Golding and Oertel, 2012; McGinley et al., 2012) and rhythmically spikes for tonal segments (see Figure 3). An ensemble of trained octopus cells executes the distributed Hough-transforms in their receptive fields. Each firing of an octopus cell indicates a found hyperbole at a specific time.

If we denote the Hough-transform by H{}, then the *r*-th row of the Hough-space (HS) patch based on the corresponding AI patch can be noted as:

(2) $$\begin{array}{l} P_{\left(u,v\right)}^{HS}\left[t,r\right]\;=\;P_{k}^{HS}\left[t,r\right]\;=\;H\left\{P_{\left(u,v\right)}^{AI}\left[t\right]\right\}\left[r\right]\;,\;\\ \;\;t\in \left\{\frac{1}{f_{s}},\;\frac{2}{f_{s}},\;\ldots ,\;\frac{N}{f_{s}}\right\}\; \end{array}$$

Pitch is ubiquitously represented by cascaded firing sweeps of octopus cells, and is derived from the global interpretation of the inter-spike interval histograms (Langner, 2015), as described in the next section.

### Pitch estimation

Every Hough-space patch is searched for minimum one and maximum three prominent curvatures *C*_*k*__, 1_, and possibly *C*_*k*__, 2_ and *C*_*k*__, 3_ (for the *k*-th HS patch), as shown in Equation (3). This is done by calculating the variance of the second order time derivative for every possible curvature row and taking the one with the highest variance value as well as maximum two more local maxima. The energy estimate in form of the RMS-value of each patch is also stored for later processing.

(3) $$\begin{array}{l} C_{k,j}=\text{arg }\underset{r}{{\text{locmax}}_{j}}\left(\underset{t}{\text{var}}(d^{2}P_{k}^{\text{HS}}[t,r]/d^{2}t)\right),\\ \;k\in \left\{1,\;2,\ldots ,\;11\right\},\;j\in \left\{1,\;2,\;3\right\} \end{array}$$

Next, each patch ${\text{P}}_{k}^{HS}$ is reduced to a sub-patch ${\overset{ˇ}{P}}_{k}^{HS}$ defined by its prominent curvature rows as indicated in the equation below:

(4) $$\begin{array}{l} {\overset{ˇ}{P}}_{k}^{HS}\left[t\right]=\left[\begin{array}{c} P_{k}^{HS}\left[t,C_{k,1}\right]\\ P_{k}^{HS}\left[t,C_{k,2}\right]\\ P_{k}^{HS}\left[t,C_{k,3}\right] \end{array}\right]\;,\;k\in \left\{1,\;2,\ldots ,\;11\right\},\;\\ \;t\in \left\{\frac{1}{f_{s}},\;\frac{2}{f_{s}},\;\ldots ,\;\frac{N}{f_{s}}\right\}\;. \end{array}$$

Each sub-patch ${\overset{ˇ}{P}}_{k}^{HS}$ undergoes an autocorrelation analysis (along the time axis). Each resulting autocorrelation function is searched for the maximum (within the lag limits deduced from *F*_*min*_ and *F*_*max*_) and from the corresponding lag the fundamental frequency ${\tilde{F}}_{k}^{\;}$ for each *k*∈{1,2, …,11} patch is estimated.

Finally, based on all the ${\tilde{F}}_{k}^{\;}$ estimates and by using the previously calculated RMS-values of the patches as weights, we calculate the (Edgeworth type) weighted median as the aggregate fundamental frequency estimate ${\tilde{F}}_{\;}^{\;}$ for the given sound snippet. This process can be seen as a weighted voting: octopus neurons belonging to each receptive field vote for their decoded fundamental frequency estimate with a weight deduced from the magnitude of the momentary micromechanical energy of the cochlear region they correspond to.

All results presented in the next chapter are supported by the above estimates.

## Results

The auditory encoder as well as the simulation of the bio-physical model of the pitch estimation have been implemented on a PC platform (in a combination of C, C++, and MATLAB languages). For evaluation and data visualization we used MATLAB from Mathworks.

### Individual sound categories

**Figure 4:**
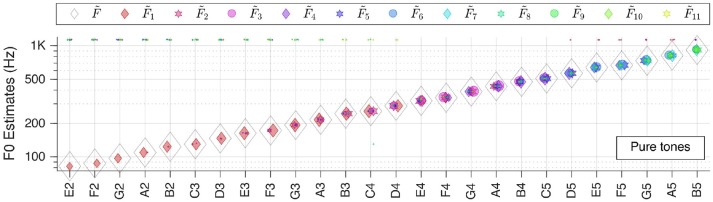
Pitch estimation performance for pure tones. On the abscissa the key tones of the sound snippets are shown in the standard scientific pitch notation. The legend applies for all figures in section Individual Sound Categories.

**Figure 5:**
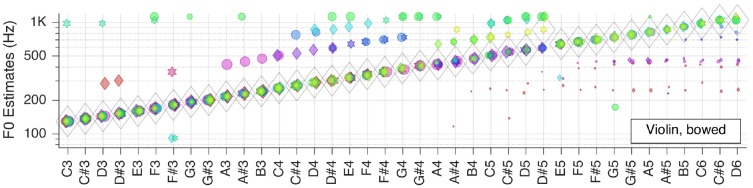
Pitch estimation performance for MUMS CD 1 Track 6 (Violin, bowed).

**Figure 6:**
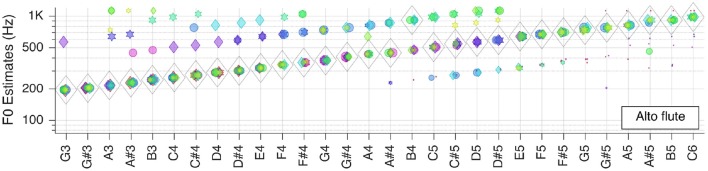
Pitch estimation performance for MUMS CD 2 Track 5 (Alto flute).

**Figure 7:**
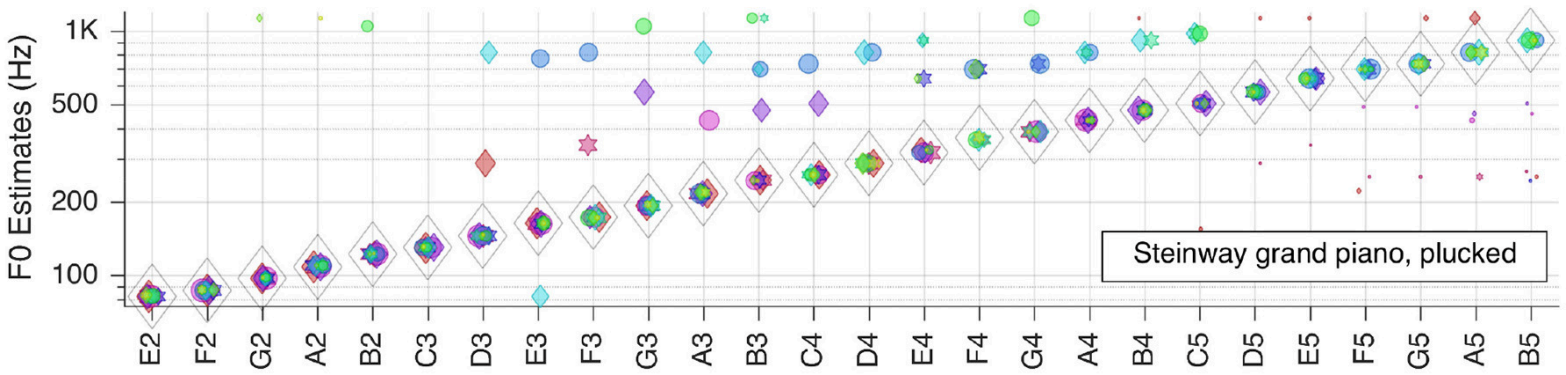
Pitch estimation performance for MUMS CD 3 Track 3 (Steinway grand piano, plucked).

**Figure 8:**
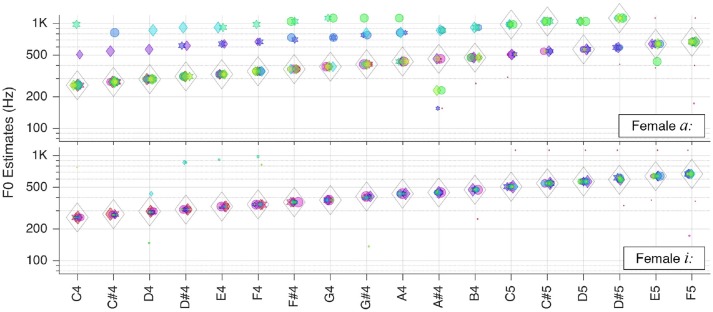
Pitch estimation performance for the vowels *a:* and *i:* sung by a female singer.

**Figure 9:**
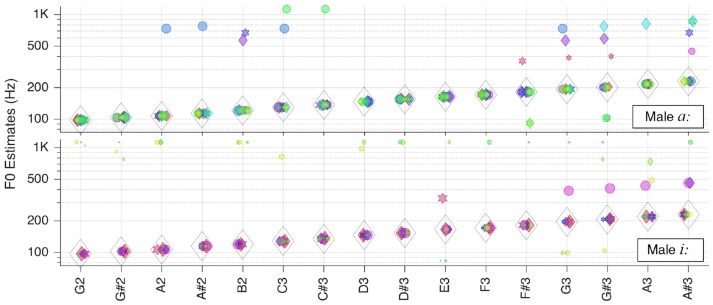
Pitch estimation performance for the vowels *a:* and *i:* sung by a male singer.

In the first instance, we tested our system with single snippets from each category (c.f. section Test Corpora and Preprocessing) at various key frequencies, at the default level of 65 dB SPL (as perceived by the auditory model), without added noise. The individual patch votes ${\tilde{F}}_{k}^{\;}$ coincide in a common ${\tilde{F}}_{\;}^{\;}$ in most cases. This ubiquitous voting scheme is robust as the majority vote counts instead of single or multiple outliers. In almost all cases the correct ${\tilde{F}}_{\;}^{\;}$ was found, as shown throughout Figures 4**–9**.

Please note that in the plots there is a linear correlation between the size of the ${\tilde{F}}_{k}^{\;}$ markers and the RMS-values of the corresponding $P_{k}^{AI}$ patches (The size of ${\tilde{F}}_{\;}^{\;}$markers is kept constant). Furthermore, some horizontal jitter has been added to the position of the markers to increase discriminability.

#### Pure tones

We first tested our system with pure sinusoidal tones to yield the ground truth for pitch estimation performance. The results are shown in Figure 4.

The performance is stable over all frequencies; ${\tilde{F}}_{\;}^{\;}$ is correct for all key tones. The figure also illustrates well that different key tones related to different cochlear regions are connected to octopus neurons of different receptive fields. And even though there are incorrect pitch votes originating from distant RFs, their weights are too low to change the final estimate.

#### Solo instruments

Next, we moved to the three selected solo musical instruments: violin, flute, and grand piano. Pitch estimation results are shown in the next three Figures 5–7, respectively.

For the bowed violin ${\tilde{F}}_{\;}^{\;}$ is correct for all but one key tone. The number of outliers is smaller for low-pitched keys, whereas there are more outliers but with smaller RMS-values in the high-pitched range.

For the alto flute ${\tilde{F}}_{\;}^{\;}$ is, again, correct for all but one key tone within the tested range. Most of the outliers seem to be attributed to octave errors during the autocorrelation step.

For the Steinway grand piano ${\tilde{F}}_{\;}^{\;}$ is correct all over the tested range of about 3.5 octaves. Most of the mid-frequency range outliers originate from one or two RFs, and indicate octave errors, but the corresponding false votes fall behind the weight of the right votes.

All in all, it can be concluded that, when only looking at the aggregate fundamental frequency estimates ${\tilde{F}}_{\;}^{\;}$, clean recordings of solo instruments lead to almost perfect pitch estimates.

#### Sung vowels

Finally, we tested the system with the sung vowels. The corresponding results are presented in Figures 8, 9.

While the female sung vowel *a:* turned out to be one of the most challenging sound in our test database, the pitch of the sung vowel *i:* could be estimated flawlessly. This shall be attributable to wider separation of the first two formants in *i:* as opposed to that in *a:*.

The situation was similar with the same vowels originating from a male singer, as shown in Figure 9. In the latter case, though, the outliers were too weak to impair pitch estimates.

### Effect of loudness

From the perspective of prevailing F0 estimators, an unusual property of our system is its sensitivity to the loudness of its input data. The employed auditory model truly mimics the essential properties of a living basilar membrane and that of the inner hair cells, so that it inherently includes various means of non-linear behavior. This also means it has, just like real ears, a sweet spot on the sound pressure level scale, where it transcodes data most faithfully.

We define gross pitch error (GPE) as the proportion of analyzed snippets, for which the relative pitch error is higher than 20%. To quantify the effects of loudness, we repeated the pitch estimation test for all 189 snippets (as presented throughout section Individual Sound Categories), but we scaled the auditory model input level in a way that the signal is “perceived” by the model at a sound pressure level between 25 and 125 dB. In practice, this means no other change to the audio signal but a linear scaling of the amplitude (with floating point precision, hence without added quantization noise).

**Figure 10:**
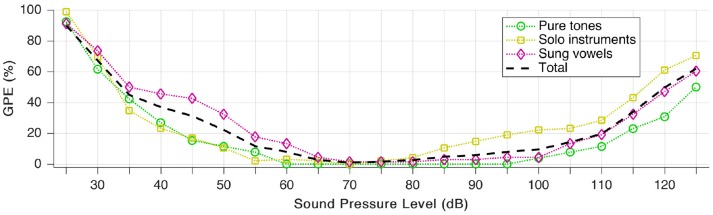
Gross pitch error (GPE) as a function of sound pressure level for the three signal categories as well as in total.

The sound pressure dependence is well-demonstrated in Figure 10. The area of the least GPE aligns well with the typical range of best speech intelligibility (Oxenham et al., 2017).

### Effect of noise

**Figure 11:**
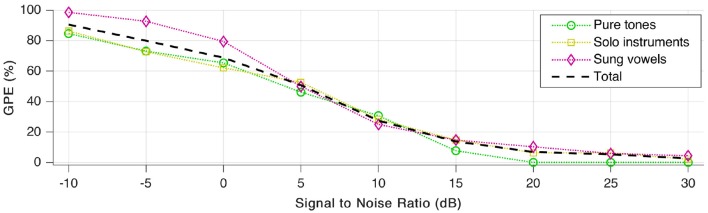
Gross pitch error (GPE) as a function of signal to noise ratio (SNR) for the three signal categories as well as in total.

Subsequently, tests were repeated a few times more, with various signal to noise ratio (SNR) values to quantify noise robustness. We added white Gaussian noise and payed attention to keep the average sound pressure level of the resulting signal at 65 dB, as was the case during our first tests without added noise. As shown in Figure 11, GPE increases rapidly with additional noise. For better than 15–20 dB SNR, the error rate decreases again, and stays low for SNR-values above 30 dB.

### Effect of snippet duration

**Figure 12:**
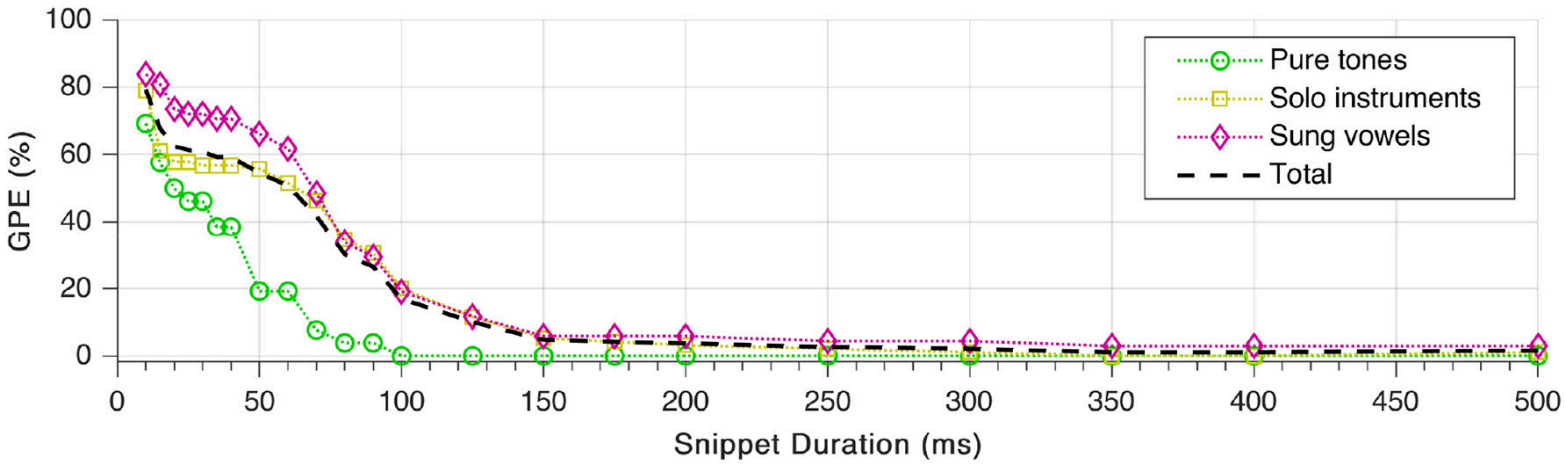
Gross pitch error (GPE) as a function of snippet duration for the three signal categories as well as in total.

Finally, we repeated the tests with different lengths of the sound snippets used for the pitch estimation. Duration of the fade-in was set to be one fifth of the total snippet duration. As shown in Figure 12, GPE decreases monotonically with increasing snippet length, which complies with expectations. The error rate starts to rise considerably only below 150 ms snippet duration. As well visible in the plot, pitches of sung vowels are the hardest to estimate correctly. What is not visible, though, that all snippets from the female sung vowel *i:* could be estimated with zero GPE down to 20 ms snippet duration.

## Discussion

We proposed a pitch estimation method based on an auditory model, with the extension of incorporating a consecutive octopus cell model. We mathematically modeled its functionality in the time domain while executing local Hough-transforms in their receptive fields to compensate for latency-phase trajectories. The model serves for explications of some aspects of neuromorphic pitch computation. Though the presented system is not yet fully mature, it is meant to pave new ways and guide the interested researcher toward new methods of pitch detection.

We tested the system with various signals over a broad semitone-scaled pitch range and saw that misclassification may occur for several reasons. It is important to mention again, that the actual pitch estimation was kept naively simple. We did not shape the autocorrelation function, nor have we used advanced statistics to enhance detection accuracy. Those means of improving the system are among our future plans. From the perspective of the simple autocorrelation back-end the misclassified outliers are very rare; hence the method has the potential to be the basis of a reliable and robust pitch estimator.

So what is our system useful for in comparison to other systems? A neuromorphic auditory system for musical notes classification has already been proposed by Cerezuela-Escudero et al. (2015). They used only a small subset of pure tones [C3, F3, C4, F4, F5, A5] and electronic piano notes [F3, F4, F5, F6]. O'Connor et al. (2013) used the neuromorphic “AER EAR” for pitch estimation, where the auditory spikes were processed with an event-based inter-spike interval histograms method. Their pure note set was limited to [A4, B4, C5, D5, E5, F5, G5#, A5, B5, C6]. The “AER EAR” as front-end and an ISIH method were used for periodicity detection in speech utterances in a limited database of speakers (Yu et al., 2009). To meaningfully compare our system to those three systems, further investigations are needed.

Our presented system can be extended to estimate multiple pitches simultaneously. This can be done either at the sub-patch (${\overset{ˇ}{P}}_{k}^{HS}$) level by substituting simple autocorrelation analysis with a multi-pitch-aware analysis method (like in Elvander et al., 2016) or in a more bio-inspired way by adding higher-level auditory functions. In the latter case, a higher auditory entity will need to reconcile the votes from all octopus cells (since in this case each of them would still only vote for one specific pitch) by sorting out false pitch votes and accepting the right ones. In such a system, decisions about wrong and right votes are based on empirical knowledge the system would need to have gathered previously, which implies the need of some kind of (machine) learning components.

Another important aspect to emphasize when evaluating the results is that we always took one snippet only (of tens to a few hundreds of ms duration) from every sound file. No information from previous or following samples within one sound file was used for the estimation of pitch. By using a sliding window with overlap to estimate pitch on a windowed basis in every sound file and relying on statistics gathered from each window, much finer and more reliable pitch estimate could be achieved (at the cost of additional computations).

SAM's auditory encoder is of type transmission-line with active outer hair cells (OHCs) numerically solved by WKB methods according to the categorization scheme of Saremi et al. (2016). The transmission line is a 1D model only (Baumgarte, 1997). OHCs are known to have piezoelectric-like properties as they have a voltage induced contractive motility (Mountain and Hubbard, 1994). The functional modeling of OHCs is recently discussed (Ó. Maoiléidigh and Hudspeth, 2015). More sophisticated 3D fluid dynamic models with interactions to the tectorial membrane and reticular lamina and active OHCs exist (Meaud and Grosh, 2012). By non-invasive volumetric optical coherence tomography in an intact cochlea of the mouse basilar membrane and tectorial membrane movements could be accurately visualized for the first time (Lee et al., 2015). A cochlear model with non-linear mechano-electrical transduction in outer hair cells can predict distortion product emissions (Liu and Neely, 2010). These findings indicate the necessity to refine the 1D model, as the vibratory patterns of the tectorial membrane influence the inner hair cell stereociliary bundles directly beneath. So SAM can be fine-tuned by explicitly modeling the tectorial membrane movement and by proper parameterization of the OHC model to correctly predict the distortion product emissions. Other instantiations of auditory models are parallel filterbanks, cascaded filterbanks, transmission-lines, and lumped-element (Lyon, 2011; Verhulst et al., 2012; Saremi and Stenfelt, 2013; Zilany et al., 2014). Their peculiarities have been systematically juxtaposed by Saremi et al. (2016). They differ in their operation as well as in their intended use. SAM, for instance, has been evaluated as the engine of a novel signal processing strategy for cochlear implants. What all these models have in common is that they all need to be customized and fine-tuned in their parameterization to become useful (Saremi and Lyon, 2018). Also, several hardware implementations of the above models exist, as for instance “AER-EAR,” “CAR-FAC” and “NAS” (Jiménez-Fernández et al., 2017; Xu et al., 2018).

We see our main contribution in demonstrating the latency-phase rectification of spatio-temporal trajectories by dendritic trees of octopus cells via modeling the executing mathematical Hough-transforms in the time domain. The beauty of the model is the ease of its predictive power, which would otherwise imply to model all rate-kinetic equations of every single cell compartment at the lower bio-physical description level (McGinley et al., 2012; Spencer et al., 2012; Wang and Liu, 2013). Furthermore, the Hough-transforms had been realized with simple shift and add operations in a grid of fixed size and implemented in hardware with simple circuit elements as flip-flops and binary adders (Epstein et al., 2002). The Hough-transform has been self-learned in a neural network and the weights converged to binary ones or zeros after the learning stage (Brückmann et al., 2004). Binary weights are advantageous for VLSI implementations as they are realizable very resource-efficiently (Bhaduri et al., 2018). For a faster convergence the Hough-transforms are numerically computed (Harczos et al., 2006).

## Conclusions

Pitch is reliably extracted over a wide range of frequency by SAM's auditory model extended by an octopus ensemble model. The model parameterization is completely described in the time domain. By using inter-spike interval histograms (ISIHs) the model is close to biological processing and therefore serves for further investigations to improve music rendering for cochlea implants.

## Author contributions

TH and FK designed and formulated the model, wrote the paper, and edited the manuscript. TH implemented and tested the model.

### Conflict of interest statement

The authors declare that the research was conducted in the absence of any commercial or financial relationships that could be construed as a potential conflict of interest. The reviewer AJ-F and handling Editor declared their shared affiliation.
